# 3D-Printed Arch Supports Combined with Toe Spreaders Modulate Phase-Specific Ankle Alignment and Muscle Activity in Young Adults with Functional Flat Foot

**DOI:** 10.3390/jcm14228017

**Published:** 2025-11-12

**Authors:** Eui-Young Jung, Shi Lei, Yujin Jeong, Hwi-Young Cho, Sanghee Park

**Affiliations:** 1 The Integrative Movement Science Laboratory, Gachon University, Incheon 21936, Republic of Korea; noel950@gachon.ac.kr (E.-Y.J.); shilei1997@gachon.ac.kr (S.L.); 17rachel@gachon.ac.kr (Y.J.); 2 Department of Physical Therapy, Gachon University, Incheon 21936, Republic of Korea; 3 Department of Exercise Rehabilitation, Gachon University, Incheon 21936, Republic of Korea

**Keywords:** functional pes planus, 3D-printed insoles, toe spreaders, ankle kinematics, electromyography

## Abstract

**Background/Objectives**: Functional flat foot (FF) is associated with altered lower limb biomechanics, leading to inefficient load transfer and potential overuse injuries. Customized orthoses, such as 3D-printed insoles and toe spreaders, may mitigate these deficits, but their combined biomechanical and neuromuscular effects remain unclear. The current study investigated the immediate effects of 3D-printed arch support insoles (SI) and toe spreaders (Toe) and their combination (SI+Toe) on gait pattern, center of force (COF), ankle alignment, and lower limb muscle activation in young adults with FF. **Methods**: Ten FF individuals and ten matched controls performed level walking under four randomized conditions: shoe-only, SI, Toe, and SI+Toe. Gait was analyzed using OptoGait, COF trajectory via F-Scan, ankle angles using Kinovea, and muscle activity (semitendinosus, biceps femoris, tibialis anterior, peroneus longus, gastrocnemius, and soleus) via surface EMG. **Results**: Compared to controls, FF individuals exhibited medial COF deviation, increased ankle eversion, and altered muscle activity. In the FF group, SI+Toe reduced medial COF deviation, decreased eversion, and prolonged foot flat while shortening the propulsive phase. Semitendinosus and tibialis anterior activity increased under SI+Toe, while gastrocnemius and soleus remained reduced during propulsion. **Conclusions**: The combined utilization of 3D-printed insoles and toe spreaders produced immediate measurable improvements in foot alignment and muscle activity patterns in FF individuals. These findings support that integrating customized orthotic designs with toe spreader elements may provide a practical, non-invasive approach for improving lower limb biomechanics. Such strategies may help improve foot mechanics and reduce compensatory muscle activation in a clinical setting.

## 1. Introduction

Functional flat foot (FF), also referred to as functional pes planus, is defined by a transient collapse of the medial longitudinal arch during weight-bearing activities, which reemerges upon unloading the foot (e.g., during rest). The altered foot posture often leads to excessive valgus stress, which disrupts the normal lower limb alignment and induces biomechanical alterations, including hindfoot pronation (a coupled motion of eversion and abduction) and compensatory forefoot supination [1,2]. Such alignment changes cause imbalanced joint loading and compensatory muscle activity, increasing the risk of musculoskeletal symptoms such as plantar fasciitis [3], ankle sprain [4], and lower limb fatigue-related pain [5].

Variations in muscle activation patterns are often observed in individuals with altered foot posture, suggesting that deficits in neuromuscular control contribute to an elevated susceptibility of lower limb injury [6,7]. Previous studies have shown that activity of extrinsic muscles during gait differs in individuals with flattened arches [8]. Muscle fatigue in the ankle and foot stabilizers further increases the likelihood of stress related injuries such as metatarsal and tibial stress fractures [9,10]. Pronated foot posture during walking, as seen in FF, altered phase-specific muscle activities. Typically, FF individuals exhibit greater activation of invertor muscles (e.g., tibialis anterior) and reduced activation of evertors (e.g., peroneus longus) compared with normally arched feet [11]. As highlighted by Murley et al., these activation differences are phase-dependent, reflecting the adaptive but inefficient neuromuscular strategies in individuals with FF. For example, tibialis anterior activity increases, and peroneus longus activity decreases during contact, whereas reduced peroneus longus activity and greater tibialis posterior activity are evident during mid-stance [6].

To mitigate these biomechanical deficits, orthotic interventions such as arch support insoles and toe spreaders have been commonly adopted [1]. These devices operate through two biomechanical strategies: direct structural support and indirect functional realignment. Direct support, exemplified by arch support insoles, counteracts midfoot collapse by applying upward force to structures such as the navicular and sustentaculum tali, thus limiting excessive eversion and redistributing the plantar load. Recent advances in 3D-printing technology enable precise customization, enhancing anatomical conformity, comfort, and plantar pressure relief in FF individuals [12,13,14,15]. Indirect realignment, as achieved by toe spreaders, targets the forefoot to influence the foot’s kinetic chain. By increasing lateral toe splay [16] and facilitating first ray mobility [17], toe spreaders enhance the windlass mechanism, thereby tightening the plantar aponeurosis and enhancing propulsion efficiency during late stance [18]. In addition, toe spreaders can modulate muscle activity, promoting balanced activation of the tibialis anterior and peroneus longus [19,20]. In summary, insoles stabilize the rearfoot and elevate the medial arch, whereas toe spreaders maintain forefoot alignment and distribute propulsive forces more evenly. The integration of additive manufacturing allows hybrid orthotic designs that combine these functions without altering normal footwear configuration [2,21,22].

Despite the demonstrated complementary mechanical benefits, prior studies have focused on isolated outcomes such as gait parameters [22], joint kinematics [2], or muscle activation [23]. Few have evaluated the integrated interaction between plantar pressure, alignment, and muscle activity. For instance, 3D-printed foot orthoses reduce peak ankle contact force and eversion moment [14,23], whereas toe spreaders improve gait stability by minimizing involuntary toe flexion and promoting more symmetrical activation of foot and leg muscle [24,25]. However, the combined biomechanical effects of both orthoses in young adults with FF remain insufficiently explored.

Thus, the present study aimed to investigate the immediate effects of 3D-printed customized arch support insoles and toe spreaders, individually and in combination, on gait patterns, COF trajectory, ankle alignment, and lower limb muscle activation in young adults with FF. We hypothesized that these interventions would produce immediate phase-specific improvements in gait patterns and ankle alignment, accompanied by more coordinated neuromuscular activation during walking.

## 2. Materials and Methods

### 2.1. Subject Information

Twenty-five individuals with potential FF were initially screened. Following evaluation, thirteen met the inclusion criteria, and three withdrew due to personal issues. Ultimately, twenty young adults participated in the current study: ten individuals with FF (5 males, 5 females; age: 21.5 ± 0.6 years; height: 167.5 ± 3.0; weight: 64.7 ± 3.3) and ten matched controls (5 males, 5 females; age: 23.0 ± 0.4 years; height: 171.1 ± 2.9 cm; weight: 71.4 ± 4.6 kg). Written informed consent form was provided to all participants prior to enrollment.

### 2.2. Identification of Functional Flat Foot

FF was diagnosed using the Foot Posture Index (FPI) in a standing position. The inclusion criteria for the FF group included (1) visible medial arch reconstitution during tiptoe standing and (2) FPI total score ≥ 6 which was assessed in the standing position. The FPI scoring was based on 6 items covering (1) talar head palpation, (2) curvature above vs. below the lateral malleoli, (3) calcaneal inversion vs. eversion, (4) level of medial arch height, (5) talonavicular congruence, and (6) forefoot abduction vs. adduction. The scores of each factor ranged from −2 to +2 (−2 for clear signs of supination, 0 for neutral, and +2 for clear signs of pronation). The total scores were summed, which ranged from −12 to +12. Subjects scoring between +6 and +10 were classified as having FF. The exclusion criteria included (1) recent use of foot orthosis in the past month, (2) surgical history involving the ankle or foot including ankle fracture/reconstruction/osteotomies, (3) known connective tissue or degenerative disorders affecting gait biomechanics such as chronic tendinopathies, plantar fasciitis, or rheumatoid arthritis, and (4) the inability to complete gait-related assessments. The average FPI score for the FF group was 7.4 ± 0.2 for both left and right feet. The study protocol was approved by the Institutional Review Board of Gachon University (IRB No. 1044396-202410-HR-171-01, November 2024), and the study adhered to the Declaration of Helsinki.

### 2.3. 3D-Printed Arch Support Insoles and Toe Spreaders

Customized arch supporting insoles were designed for each participant using a 3D scanner (Shining 3D, San Leandro, CA, USA) to capture the precise representation of the foot’s shape. Designs were then created by reverse engineering techniques with Autodesk Fusion 360 (Autodesk Inc., San Francisco, CA, USA), producing an insole that extended approximately two-thirds of the total foot length, terminating approximately 10% (of total foot length) proximal to the first metatarsal head and approximately 4% (of total foot length) proximal to the fifth metatarsal head [14] (Supplementary S1a,c). The insole incorporated a 2.5 mm base thickness and a U-shaped rearfoot contour with a flat base to cradle the calcaneus and minimize eversion, providing rearfoot stabilization. The midfoot region incorporated a contoured medial arch elevation (approximately 8 to 10 mm depending on foot morphology) to restore navicular height and distribute load across the medial longitudinal arch. The final product was printed using fused deposition modeling technology with PLA filament (diameter: 1.75 mm; nozzle temperature: 210 °C; layer thickness: 0.2 mm; infill: 20%; print speed: 120 mm/s), requiring approximately four hours per insole. A 1 mm polyamide nylon cushion layer was attached to enhance comfort and shock absorption (Supplementary S1b). Commercially available silicone toe spreaders (Golbylicc, Portland, OR, USA) were used to assess forefoot alignment effects (Supplementary S1d). The toe spreaders were positioned between each of the first through fifth toes, promoting inter-metatarsal spacing and repositioning the metatarsophalangeal joints toward neutral transverse width. The configuration was intended to enhance tension along the plantar fascia, supporting load transfer from the lateral heel to the first and second metatarsal heads during propulsion [26]. Together, the insoles and toe spreaders were designed to assist in stabilizing the calcaneus, elevate the medial arch, and maintain functional toe alignment throughout the stance phase.

### 2.4. Experimental Procedures

Gait assessment was conducted in the Integrative Movement Science Laboratory at Gachon University. To gather kinematic and kinetic data, the following tools were used: Optogait (version 1.13.24.0) (Microgate, Bolzano, Italy) for gait pattern assessment, F-Scan (version 6.85) (TekScan, Norwood, MA, USA) for center of force measurements, Kinovea (version 2023.1.2) for ankle alignment analysis, and surface EMG (Biopac System, Goleta, CA, USA) to monitor muscle activity pattern. Participants attended three visits. The first visit included baseline measurements (e.g., FPI assessment, anthropometrics, and foot scanning). The second visit involved the customized insole fitting, comfort check, and initial familiarization session. In the third visit, experimental gait trials for each test condition were conducted after a 5 min familiarization session per condition. Each subject walked at a self-selected speed under four conditions: (1) shoe only (control or FF), (2) shoe with 3D-printed insoles (SI), (3) shoe with toe spreaders (Toe), and (4) combination of insoles and toe spreaders (SI+Toe). All participants wore flat cushioned sneakers (Converse-style; JK Company, Gimhae, Republic of Korea) approximately 1 cm larger than their usual size to avoid the mechanical restriction of toe splay, following recommendations from a previous toe spreader validation study [27]. To ensure that the toe spreader was the only independent variable, the same footwear size was consistently used across all conditions. The condition order was determined through block randomization (block size = 4) to minimize potential ordering effects.

### 2.5. Outcome Measurement

#### 2.5.1. Gait Pattern Analysis

Spatiotemporal gait parameters were captured using the OptoGait system (Microgate, Bolzano, Italy). Two 1 m sensor bars, each equipped with 96 infrared LEDs, were mounted on the sides of a treadmill, 2 mm above floor level. Three stance phases (contact, foot flat, and propulsive) and the swing phase were identified based on LED interruptions during walking trials on a flat surface. Data were sampled at 1000 Hz. According to the OptoGait manufacturer’s guidelines, the contact phase was defined as the period from initial heel strike to the moment when the entire plantar surface first touched the ground. The foot flat phase extended from full plantar contact to heel-off, representing the mid-stance period in which the body weight was transferred to the supporting foot. The propulsive phase began heel-off to toe-off, corresponding to the terminal stance when the forefoot generated forward propulsion. The swing phase contains the non-contact phase between toe-off and the subsequent heel strike. Previous studies have demonstrated that the use of insoles can influence the plantar pressure distribution by increasing the loading of the second to fourth metatarsal regions and enhancing the midfoot contact area, thereby producing notable alterations in the foot flat and propulsive phases [22]. Based on the evidence, the current study was focused on the analysis of three key spatiotemporal parameters: the contact, foot flat, and propulsive phases, while adding the swing phase for muscle activity analysis, due to important evidence showing that increased stability by foot orthosis can change muscle activity during single leg support [28].

#### 2.5.2. Center of Force Analysis

COF trajectories were analyzed using the F-Scan out-shoe system (TekScan Inc., Norwood, MA, USA). Although the F-scan is conventionally used in an in-shoe configuration, the out-shoe setup was adopted in the current study to preserve the natural interaction between the foot, the 3D-printed insoles, and the toe spreaders without altering the footwear fit or internal alignment. The primary methodological considerations for the out-shoe configuration were ensuring stable sensor fixation. To achieve this, adhesive tapes were applied to secure the F-scan sensors firmly on the shoe surface at the anterior and posterior regions, first and fifth metatarsal head regions, and medial and lateral regions of the calcaneus. The system continuously captured the dynamic plantar pressure distribution and COF shifts from at least 10 consecutive gait cycles per trial, enabling real-time tracking of medio-lateral and antero-posterior COF dynamics during walking.

#### 2.5.3. Ankle Alignment Analysis

Frontal-plane ankle alignment was analyzed using a two-dimensional video-based approach. Three reflective markers were placed on the posterior calcaneus (on the shoe surface), posterior talus, and midpoint of the gastrocnemius. Rear-view recordings were obtained (30 fps) at ankle height using a digital camera (NaturalPoint Inc., Corvallis, OR, USA). Frontal-plane inversion/eversion angles were analyzed in Kinovea (version 2023.1.2) [29]. Ankle angles were calculated as the angle formed between a line connecting the posterior talus and calcaneal makers and a vertical reference line passing through the gastrocnemius marker. Positive values indicated eversion, and negative values indicated inversion. Mean ankle angles were averaged from at least 10 consecutive strides per condition.

#### 2.5.4. Electromyographic Recording

A wireless surface EMG was utilized using a seven-channel EMG system (BIOPAC MP160; BIOPAC Inc., Goleta, CA, USA) (CMRR: >110 dB at 60 Hz, input impedance > 10^12^ Ω, gain: 2000 and band-pass filter: 20 Hz low cut-off, 500 Hz high cut-off). The collected surface EMG signals were processed using Acknowledge 5.0 software (Biopac Systems, Inc., Goleta, CA, USA). Disposable electrodes (Bio-Protech Inc., Tustin, CA, USA) (diameter: 10 mm, bipolar configuration, and interelectrode distance > 20 mm) were used. Electrodes were positioned on the muscle bellies of the lower leg muscles (dominant leg) in accordance with SENIAM guidelines on seven muscles: tibialis anterior, peroneus longus, soleus, gastrocnemius medialis, gastrocnemius lateralis, biceps femoris, and semitendinosus. A ground electrode was placed on the subject’s ankle (both medial and lateral malleolus) and tibial tuberosity. To reduce movement artifacts, electrode cables were tightly secured to the skin using elastic tapes. The EMG signals were collected at a sampling frequency of 1000 Hz. The raw EMG data were processed: full-wave rectification and the root mean square smoothing (50 ms moving window). Each condition was analyzed using six gait cycles. Muscle activity amplitudes during each phase of the gait cycle were normalized to the internal mean of the respective muscle activity across the entire gait cycle rather than to the maximal voluntary isometric contraction (MVIC). The internal mean normalization method, recommended for dynamic gait analysis and described in previous studies [30,31], reduces interindividual variability arising from day-to-day or trial-to-trial fluctuations in voluntary contraction effort. Because gait is a highly cyclical task with greater intra-cycle variation than other exercise modalities, the approach provides more consistent comparisons of phase-specific activation patterns. The gait cycle from heel strike to the end of swing phase was firmly confirmed by using DTS footswitch (Noraxon, Scottsdale, AZ, USA) synchronized with EMG activity in the target muscles following the commercially available protocol. Since the gait cycle contains a different number of samples within a given gait sequence, we applied time normalization by linear interpolation to 101 for each cycle [31]. Each data sample underwent identical processing steps, and the resulting values were averaged across each phase of the gait cycle.

### 2.6. Statistical Analyses

The sample size was estimated using G*Power (v3.1; Universität Kiel, Kiel, Germany) for an effect size of 0.5, alpha = 0.05, and power = 0.80, resulting in a required sample of 20, which aligns with prior biomechanical studies of similar design [13,32]. The selected medium effect size (Cohen’s d = 0.5) was justified based on previous orthotic and gait biomechanics studies which reported comparable magnitudes of changes in ankle alignment, plantar pressure, or EMG activation following orthotic interventions. Normality was confirmed with the Shapiro–Wilk test. Control conditions involving orthotic applications (CTR_SI, CTR_Toe, and CTR_SI+Toe) were excluded from the main comparative analyses to maintain clarity and focus on the primary hypothesis concerning the FF group. Thus, a mixed effects model using restricted maximum likelihood estimation was applied, instead of a traditional repeated measures ANOVA followed by Holm–Sidak’s multiple comparisons test applied by within-measure but not across-outcomes correction. The model included two fixed factors, group (control vs. FF) and condition (shoe-only, SI, Toe, and SI+Toe) to evaluate differences in gait patterns, COF, joint alignment, and muscle activity. To control for multiple testing, Holm–Sidak correction was applied within each outcome family. When applicable, targeted comparisons were additionally assessed using paired *t*-tests (Microsoft Excel, Microsoft Corporation, San Francisco, CA, USA). GraphPad Prism (version 10.3.1; GraphPad Software, San Diego, CA, USA) was used for all statistical analyses. Data were presented as the mean ± standard error of the mean (SEM). A significance level of *p* < 0.05 was applied to all statistical tests.

## 3. Results

The outcomes were analyzed across multiple factors including the gait pattern, COF, ankle alignment, and lower limb muscle activation. Gait variables were examined in relation to the stance phase divided into contact, foot flat, and propulsive phases. Muscle activation pattern was assessed throughout the entire gait cycle, consisting of the stance phase (contact, foot flat, and propulsive phases) and the swing phase.

### 3.1. Gait Pattern

**Figure 1:**
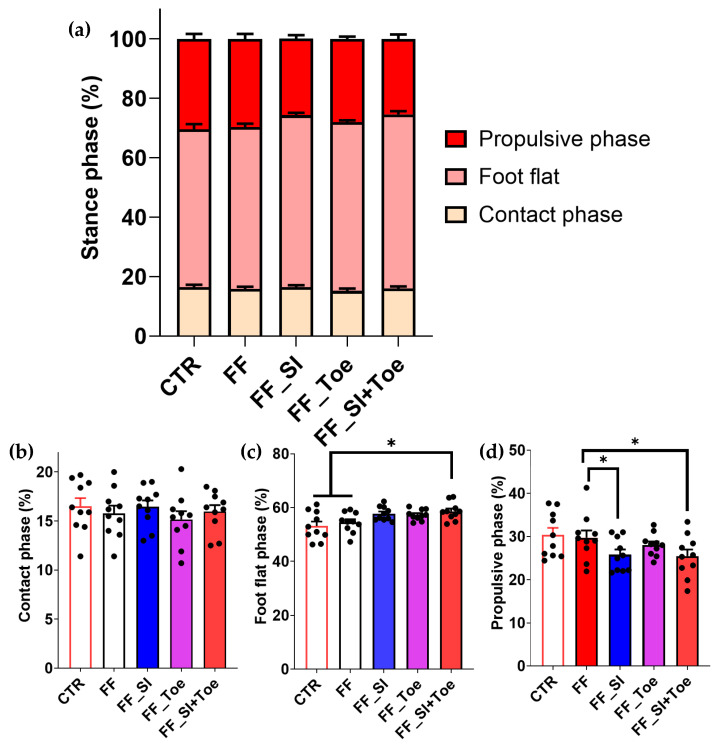
Gait pattern analysis in the comparison of the stance phase components. (**a**) Stance phase across conditions, (**b**) contact phase duration (heel strike to foot flat), (**c**) foot flat phase duration (full foot contact), (**d**) propulsive phase duration (heel lift to toe-off). Significant main effects (*p* < 0.05) were observed between interventions in foot flat and propulsive phases; * indicates significant differences between the indicated comparison. CTR: control, FF: functional flat foot.

Gait pattern analysis revealed no significant group differences in the contact phase (Figure 1b). However, in individuals with FF, the duration of the foot flat phase was increased in the SI+Toe condition compared to the shoe-only condition (*p* < 0.05) (Figure 1c). The propulsive phase was decreased in the SI and SI+Toe conditions compared to the shoe-only condition (*p* < 0.05) (Figure 1d).

### 3.2. Center of Force

**Figure 2:**
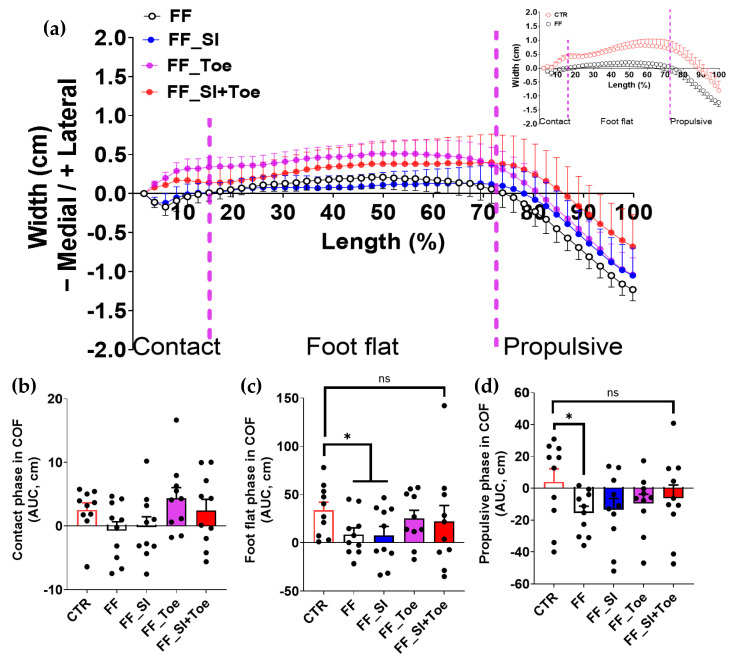
Averaged center of force (COF) trajectory during level walking in individuals with FF under four orthotic conditions in the comparison of the stance phase components. (**a**) Lateral–medial COF displacement throughout stance (0–100%), divided into contact (0–16%), foot flat (16–73%), and propulsive (73–100%) phases. Positive values indicate lateral deviation; negative values indicate medial deviation. The inset compares CTR and FF in the shoe-only condition. (**b**–**d**) AUC of COF displacement for each stance sub-phase: (**b**) contact, (**c**) foot flat, and (**d**) propulsive. Significant main effects (*p* < 0.05) were observed between CTR and FF groups in foot flat and propulsive phases. Dashed vertical lines indicate averaged phase boundaries. AUC quantifies the extent of medial–lateral COF displacement for each phase. * denotes statistically significant differences between the indicated comparison; ns: not significant. AUC: area under the curve, CTR: control, FF: functional flat foot.

Analysis of the COF trajectory during the stance phase showed a greater medial deviation during the foot flat and the propulsive phases in the FF group compared to the controls (*p* < 0.04), while the contact phase revealed no significances between the groups (Figure 2). Notably, the SI+Toe condition in FF individuals exhibited a lateral shift during the foot flat phase, aligning closely with that in the control (Figure 2c). During the propulsive phase in the FF individuals, the SI+Toe condition induced a lateral displacement, resulting in statistically indistinguishable COF patterns from controls (Figure 2d).

### 3.3. Ankle Alignment

**Figure 3:**
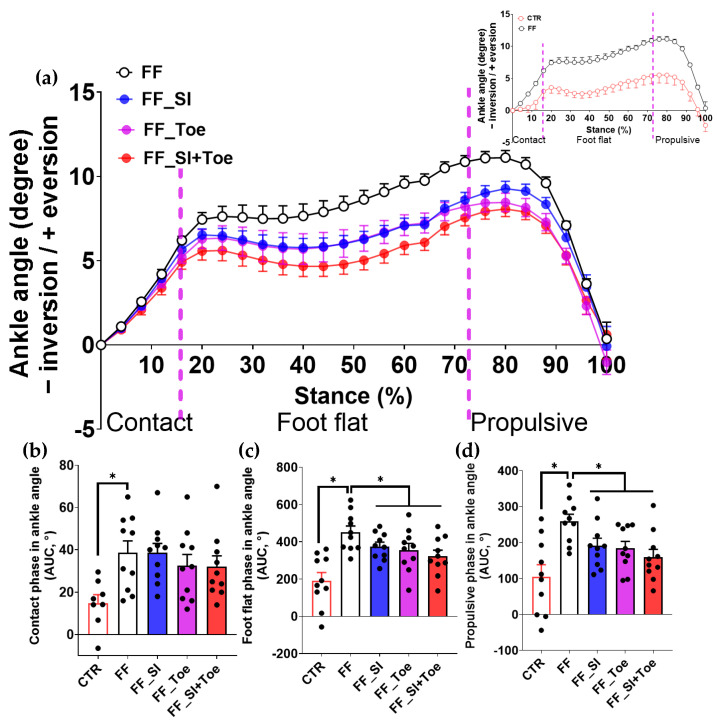
Ankle joint angles (frontal plane) during level walking in the comparison of the stance phase components. (**a**) Averaged ankle angles (eversion [+]/inversion [−]) across stance (0–100%), segmented into contact (0–16%), foot flat (16–73%), and propulsive (73–100%) phases. The inset compared CTR and FF under the shoe-only condition. (**b**–**d**) AUC of ankle joint angles for each stance subphase: (**b**) contact, (**c**) foot flat, (**d**) propulsive. Dashed vertical lines indicate averaged phase boundaries. Significant main effects (*p* < 0.05) were observed between CTR and FF groups in contact phase and between interventions in foot flat and propulsive phases; * indicates significant differences between the indicated comparison. AUC: area under the curve, CTR: control, FF: functional flat foot.

Ankle angle analysis in the frontal plane revealed that ankle eversion was significantly increased in FF individuals across all stance sub-phases compared to controls (*p* < 0.01), most notably during foot flat and propulsion (*p* < 0.001) (Figure 3). During the foot flat and the propulsive phases, all interventions significantly reduced eversion angles compared to the FF shoe-only condition (*p* < 0.05), with the SI+Toe condition yielding ankle kinematics most closely resembling those of the control group (Figure 3c,d).

### 3.4. Muscle Activity

**Figure 4:**
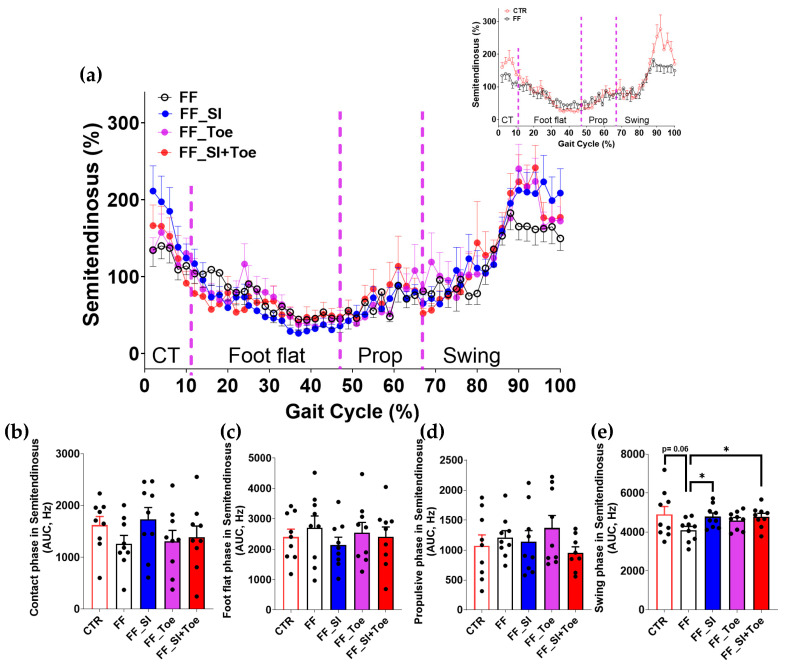
Semitendinosus muscle activation during level walking. (**a**) Averaged linear envelope EMG (% mean EMG activities within condition) across the gait cycle, segmented into contact, foot flat, propulsive, and swing phases. The inset compares CTR and FF under the shoe-only condition. Dashed vertical lines indicate average phase boundaries. (**b**–**e**) AUC of semitendinosus activation for each phase: (**b**) contact, (**c**) foot flat, (**d**) propulsive, and (**e**) swing. A significant main effect (*p* < 0.05) was observed between interventions in swing phase; * indicates significant differences between the indicated comparison. AUC: area under the curve, CTR: control, FF: functional flat foot.

**Figure 5:**
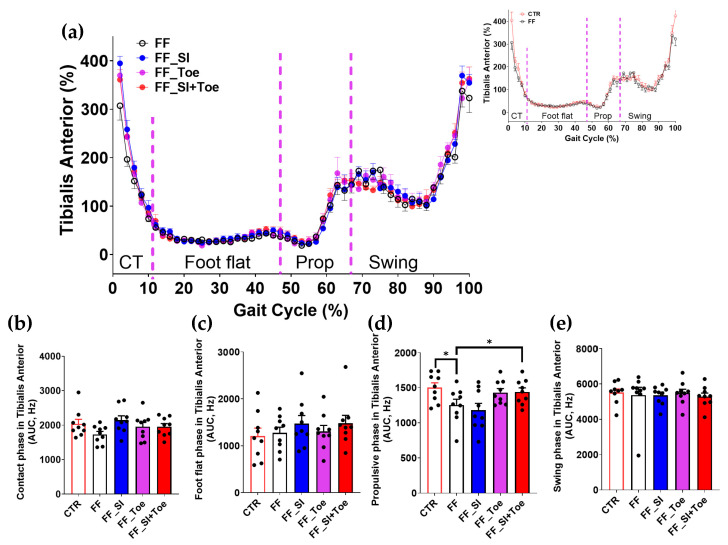
Tibialis anterior muscle activation during level walking. (**a**) Averaged linear envelope EMG (% mean EMG activities within condition) across the gait cycle, segmented into contact, foot flat, propulsive, and swing phases. The inset compares CTR and FF under the shoe-only condition. Dashed vertical lines indicate averaged phase boundaries. (**b**–**e**) AUC for tibialis anterior activation in each phase: (**b**) contact, (**c**) foot flat, (**d**) propulsive, and (**e**) swing. Significant main effects (*p* < 0.05) were observed between CTR and FF groups and between interventions in propulsive phase; * indicates significant differences between the indicated comparison. AUC: area under the curve, CTR: control, FF: functional flat foot.

**Figure 6:**
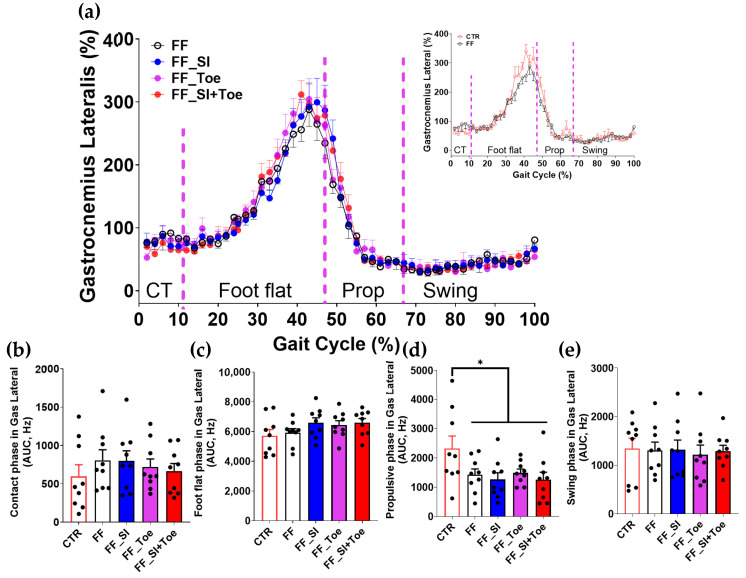
Gastrocnemius lateralis muscle activation during level walking. (**a**) Averaged linear envelope EMG (% mean EMG activities within condition) across the gait cycle, segmented into contact, foot flat, propulsive, and swing phases. The inset compares CTR and FF under the shoe-only condition. Dashed vertical lines indicate averaged phase boundaries. (**b**–**e**) AUC for gastrocnemius lateralis activation in each phase: (**b**) contact, (**c**) foot flat, (**d**) propulsive, and (**e**) swing. A significant main effect (*p* < 0.05) was observed between CTR and FF groups in propulsive phase; * indicates significant differences between the indicated comparison. AUC: area under the curve, CTR: control, FF: functional flat foot.

**Figure 7:**
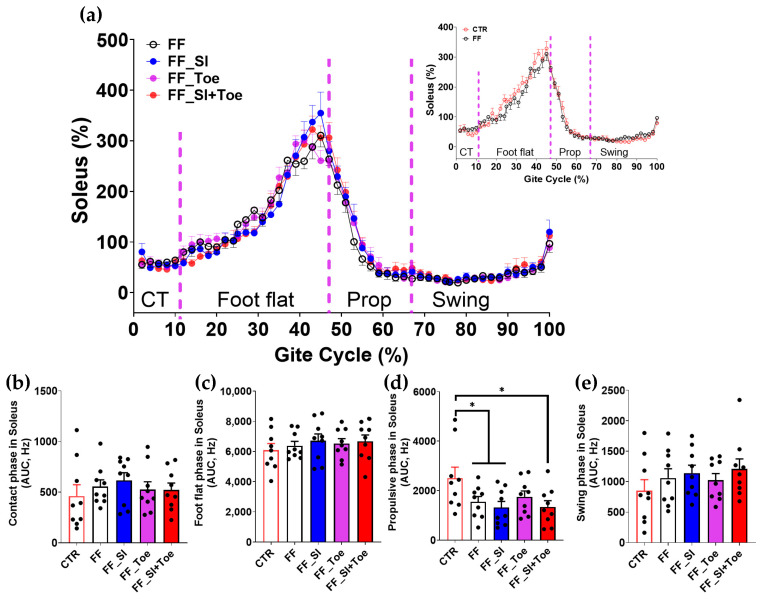
Soleus muscle activation during level walking. (**a**) Averaged linear envelope EMG (% mean EMG activities within condition) across the gait cycle, segmented into contact, foot flat, propulsive, and swing phases. The inset compares CTR and FF under the shoe-only condition. Dashed vertical lines indicate averaged phase boundaries. (**b**–**e**) AUC for soleus activation in each phase: (**b**) contact, (**c**) foot flat, (**d**) propulsive, and (**e**) swing. A significant main effect (*p* < 0.05) was observed between CTR and FF groups in propulsive phase; * indicates significant differences between the indicated comparison. CTR: control, FF: functional flat foot, AUC: area under the curve.

**Figure 8:**
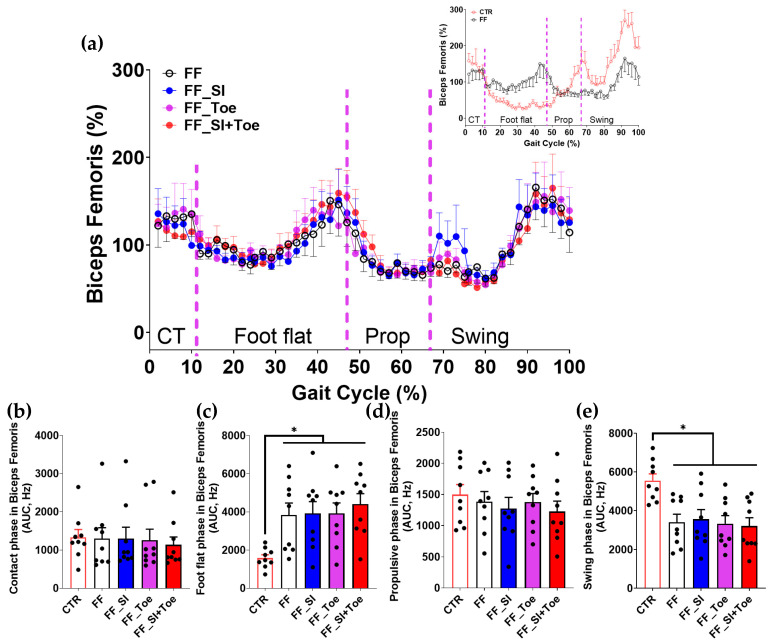
Biceps femoris muscle activation during level walking. (**a**) Averaged linear envelope EMG (% mean EMG activities within condition) across the gait cycle, segmented into contact, foot flat, propulsive, and swing phases. The inset compares CTR and FF in the shoe-only condition. Dashed vertical lines indicate averaged phase boundaries. (**b**–**e**) AUC for biceps femoris activation in each phase: (**b**) contact, (**c**) foot flat, (**d**) propulsive, and (**e**) swing. Significant main effects (*p* < 0.05) were observed between CTR and FF groups in the foot flat and swing phases; * indicates significant differences between the indicated comparison. AUC: area under the curve, CTR: control, FF: functional flat foot.

Analysis of the muscle activity revealed phase-dependent changes across the gait cycle (Figure 4, Figure 5, Figure 6, Figure 7 and Figure 8). In the semitendinosus, the swing phase activity tended to be reduced under the FF shoe-only condition compared to the control (*p* = 0.06) but was significantly increased with the SI and SI+Toe (*p* < 0.05) (Figure 4e). Tibialis anterior activation was suppressed in FF individuals during the propulsive phase under the shoe-only condition compared to controls (*p* < 0.05), whereas the SI+Toe restored activation levels in the propulsive phase (*p* < 0.02) (Figure 5d). Gastrocnemius lateralis and soleus activity declined during the propulsive phase in all FF interventions (*p* < 0.05), except for a non-significant trend with the Toe condition in soleus (*p* < 0.15) (Figure 6d and Figure 7d). Lastly, biceps femoris activity was significantly alternated during the foot flat and swing phases across all FF conditions compared to the control (*p* < 0.05) (Figure 8c,e). No significant differences were observed in gastrocnemius medialis and peroneus longus activity under any conditions (Supplementary S3 and S4).

## 4. Discussion

The current study demonstrated the immediate effects of 3D-printed arch support insoles and toe spreaders, both individually and in combination, on gait patterns, COF trajectory, ankle alignment, and lower limb muscle activation in young adults with FF. Our results confirmed that both arch supported insoles and toe spreaders modified COF and ankle alignment, with greater adjustments under the combined intervention. These findings support the complementary roles of rearfoot and forefoot realignment. Notably, semitendinosus and tibialis anterior activity showed significant modulation, indicating improved neuromuscular coordination.

### 4.1. Modification of COF Trajectory

FF individuals showed a medial shift in the COF during foot flat and propulsion (Figure 2), consistent with previous findings of excessive eversion and disrupted load transfer in FF [23]. Our findings showed that the toe spreaders and more prominently the combined SI+Toe condition redirected the medial shift toward a laterally balanced COF trajectory similar to control. These results align with prior studies demonstrating that toe spreaders realigned metatarsophalangeal joints to a neutral position and mildly extended interphalangeal joints, reducing toe flexion during stance and propulsion [24,25]. This restored normal plantar contact and load transfer, supporting plantar fascia tension and the medial arch elevation during propulsion. Consequently, the COF followed a physiologically balanced path, consistent with Bojsen–Møller’s windlass mechanism [26]. Additionally, the base of support, defined as the area of contact between the foot and the ground, was altered by using the toe spreaders. Expanding the forefoot’s contact area also increased the base of support stability, enhancing the medial–lateral balance. Similarly, Lin et al. showed increased lateral COF deviation with 3D-printed orthoses [13]. Taken together, these results imply that the concurrent application of arch support and toe spreaders may provide enhanced correction of medial deviation commonly observed in FF during stance.

### 4.2. Reduction in Ankle Eversion

Both the arch support insoles and toe spreaders reduced excessive ankle eversion during foot flat and propulsion (Figure 3). These findings align with previous studies showing that arch support orthoses reduced eversion and improved subtalar joint stability [14,33], while toe spreaders improved hallux valgus alignment in the pronated foot [34]. Although the additive effects were modest, the combined intervention achieved the most stable ankle alignment across stance. Rearfoot stabilization and forefoot expansion appeared to work synergistically to enhance overall ankle alignment during gait cycle. Furthermore, reduced pronation has been associated with lower injury susceptibility and better postural control [35,36,37], supporting the clinical potential of dual orthotic strategies in FF management.

### 4.3. Phase-Specific Changes in Muscle Activation

Lower limb muscle activity showed phase-dependent adaptations to orthotic interventions. Semitendinosus activity, reduced during swing in FF, was restored under SI and SI+Toe (Figure 4), suggesting improved knee stability and valgus control [38]. These results are consistent with prior findings that orthoses can modify proximal muscle recruitment in response to altered foot alignment [39]. Tibialis anterior activation, reduced during propulsion in FF, was restored in SI+Toe (Figure 5), indicating enhanced dorsiflexor control. However, these results contrast with previous studies showing higher tibialis anterior activity in FF during contact [6,40]. The differences likely stem from the EMG normalization methods and testing conditions. The current study normalized EMG signals to the internal means rather than MVIC, which can yield different amplitude magnitudes. Specifically, given that MVIC values are higher in control participants, normalization to MVIC may underestimate EMG values in controls, a point supported by the previous literature on strength-normalized workloads [41]. Additionally, trials in the current study were conducted under shod conditions, whereas the previous study utilized barefoot walking, influencing muscular stabilization strategies. Murley et al. reported altered tibialis anterior activation in FF when wearing shoes and orthosis compared to barefoot walking [42]. Moreover, the reduced tibialis anterior activation observed in our FF participants is consistent with prior studies suggesting impaired dorsiflexor function during walking in FF [43,44]. The insoles in the present study, featuring reinforced rearfoot support and individualized arch height, may have promoted dorsiflexor engagement. Despite these improvements, gastrocnemius and soleus activity remained reduced during propulsion across all conditions (Figure 6 and Figure 7), suggesting that short-term orthotic application may be insufficient to restore plantarflexion function in FF [40]. Collectively, SI+Toe produced the most normalized muscle activation pattern, emphasizing the advantage of combining structural and functional realignment.

From a clinical perspective, the current findings indicate that physiotherapists or clinicians can achieve rapid improvements in gait efficiency and lower limb alignment by prescribing combined 3D-printed arch support insoles and toe spreaders. The SI+Toe configuration may be particularly beneficial in early rehabilitation, where enhancing dorsiflexor and medial hamstring activation is essential for restoring dynamic knee and ankle control. In practice, clinicians may incorporate such orthotic devices alongside gait retraining protocols or progressive loading exercises to improve neuromuscular coordination and reduce compensatory overpronation in FF. Such integrated interventions are cost-effective, easily customized, and applicable in both preventative and therapeutic settings.

### 4.4. Residual and Unexpected Effects

Despite improvements in foot alignment and muscle activation, residual asymmetries persisted. Biceps femoris showed increased activity during foot flat and reduced activation during swing across all FF conditions (Figure 8). This may reflect intrinsic adaptations unresponsive to short-term interventions. Although gait pattern parameters remained unchanged, orthotic interventions prolonged foot flat and reduced propulsion (Figure 1). This shift may indicate load redistribution and reduced propulsive demand. The shortened propulsive phase may reflect enhanced forward momentum efficiency, aligning with earlier studies showing enhanced force transfer after orthotic use [22,45,46,47]. Overall, our findings indicate that compensatory muscle response may require extended adaptation for complete normalization. Nonetheless, the observed adjustments indicate a beneficial adaptation toward more efficient gait mechanics.

### 4.5. Limitations

Several limitations should be acknowledged. First, the current study included only young adults; so, the findings cannot be generalized to older or clinical populations. Second, as the study examined immediate rather than long term effects, potential adaptation or retention responses that may occur with long term orthotic application were not evaluated. Accordingly, sustained adaptations with extended orthotic use need to be established to determine whether the observed acute improvements are maintained or enhanced over time. Third, ankle kinematics in the current study were captured with a 2D video-based method rather than full 3D motion capture (e.g., Vicon or Opti Track). Although a 3D system would enable detailed analysis of multiplanar motion, the primary focus of this investigation was on phase-specific muscle activation and frontal plane alignment, which can be accurately quantified using validated 2D techniques. The use of 2D analysis was therefore considered methodologically appropriate given the available resources and the current study’s primary objectives. Despite these limitations, the current study provides meaningful insight into the acute biomechanical responses to combined 3D-printed orthotic and toe alignment interventions in FF.

## 5. Conclusions

In conclusion, the present study demonstrated that 3D-printed customized arch support insoles and toe spreaders, applied individually or in combination, acutely improved foot biomechanics in young adults with FF. The combined application resulted in a consistent reduction in medial COF deviation and ankle eversion during foot flat and propulsive phases. Furthermore, muscle activation patterns showed phase-specific modulation, with greater semitendinosus and tibialis anterior activity under the combined condition, suggesting improved dynamic limb control. Nevertheless, persistent attenuation in plantar flexor muscles implies that short-term orthotic usage may not immediately restore chronically altered muscle function. Additionally, adaptations in gait timing, including a longer foot flat duration and shorter propulsion, reflect an acute redistribution of plantar load and adaptation to orthotic use. Taken together, integrating rearfoot stabilization with forefoot realignment through customized orthotic design provides distinct acute biomechanical benefits over single-device applications. These immediate improvements may provide a basis for long-term neuromuscular adaptation through continued orthotic use and gait retraining. Future longitudinal studies are warranted to determine whether these acute effects translate into sustained functional gains and inform optimized orthotic and rehabilitation strategies for individuals with FF.

## Data Availability

The original contributions presented in this study are included in the article/Supplementary Materials. Further inquiries can be directed to the corresponding authors.

## Supplementary Materials

The following supporting information can be downloaded at: https://www.mdpi.com/article/10.3390/jcm14228017/s1, Figure S1: Design and application process of 3D-printed arch support insoles and toe spreaders; Figure S2: Representative experimental setup for gait analysis; Figure S3: Gastrocnemius medialis muscle activation during level walking; Figure S4: Peroneus longus muscle activation during level walking.
