# Supplementary material for: 3D-Printed Arch Supports Combined with Toe Spreaders Modulate Phase-Specific Ankle Alignment and Muscle Activity in Young Adults with Functional Flat Foot
Source: J Clin Med. 2025 Nov 12;14(22):8017. doi: 10.3390/jcm14228017 (PMC12653742; doi:10.3390/jcm14228017)
Supplement: Supplementary file 1 [file jcm-14-08017-s001.zip › jcm-3970516-supplementary.pdf]

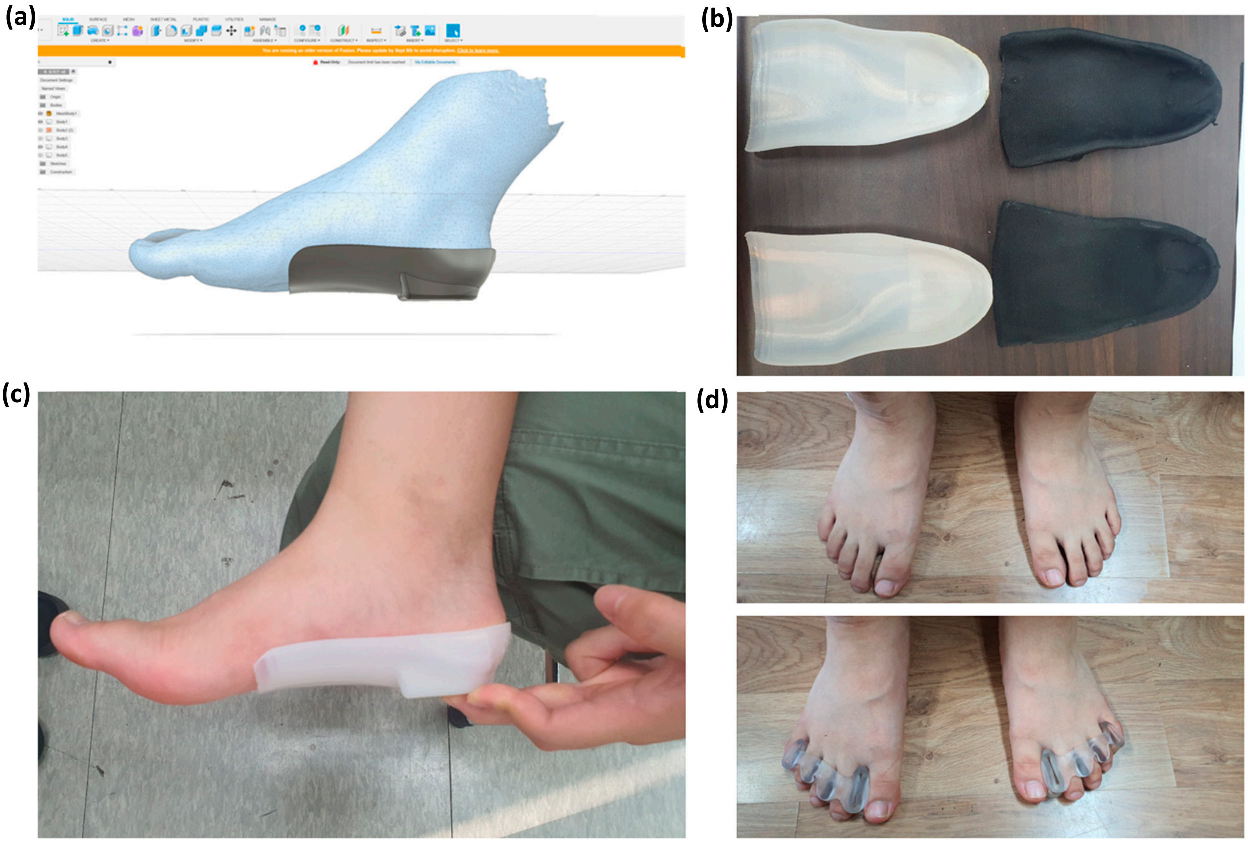

Supplementary 1. Design and application process of 3D-printed arch support insoles and toe spreaders. (a) Digital modeling of the insole using reverse engineering techniques in Autodesk Fusion 360, which is based on a 3D scan of the plantar surface. (b) Final printed insoles (left) and insoles with applied nylon cushion cover (right), shown from a superior view. (c) Fitting of the 3D-printed insole to the participant's foot to verify anatomical conformity and comfort prior to experimental testing. (d) Application of silicone toe spreaders during standing, demonstrating baseline (top) and intervention (bottom) foot posture.

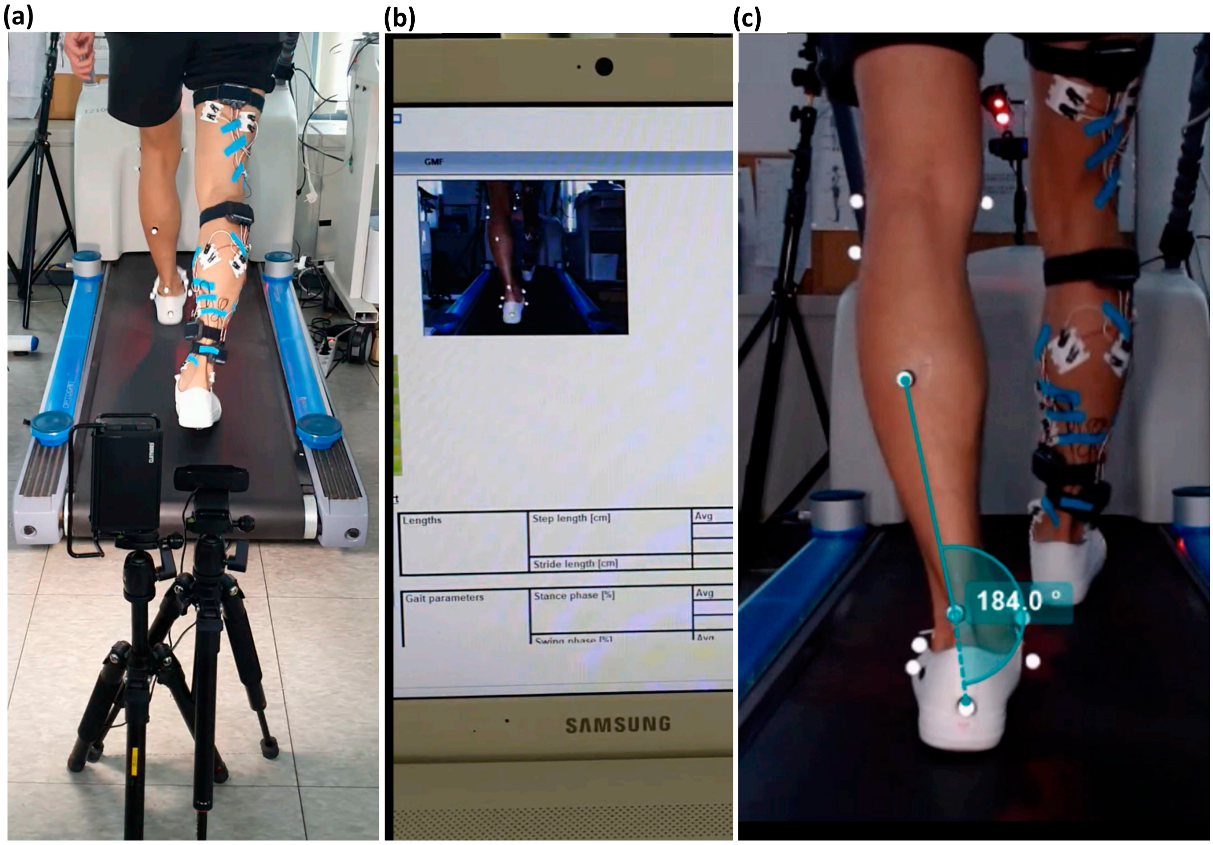

Supplementary 2. Representative experimental setup for gait analysis. (a) Overall setup showing a participant walking on a treadmill equipped with OptoGait infrared sensor bars for spatiotemporal gait analysis, surface EMG system for lower limb muscle activity recording, reflective markers for ankle alignment tracking, and a rear-view camera positioned for kinematic capture. (b) Real-time video displayed on the analysis computer, showing synchronized recordings from the rear-view camera and OptoGait recordings during walking trials. (c) Example of frontal plane ankle alignment analysis performed in Kinovea software, using markers placed on the posterior talus, calcaneus, and at the lowest point between the lateral and medial gastrocnemius borders to calculate eversion-inversion angles throughout the gait cycle.

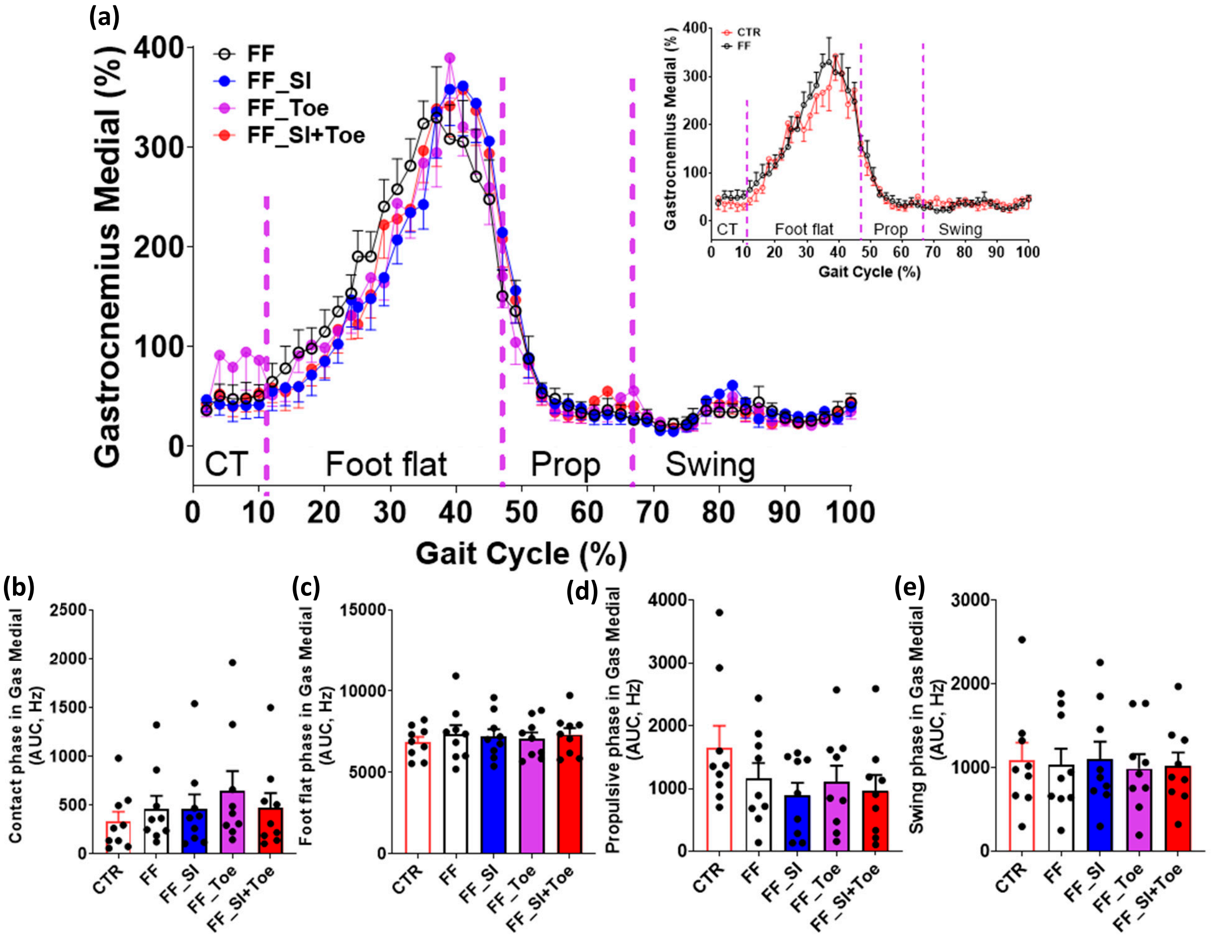

Supplementary 3. Gastrocnemius medialis muscle activation during level walking. (a) Averaged linear envelope EMG activity (% of mean EMG activities in each condition) across the gait cycle, segmented into contact, foot-flat, propulsive, and swing phases. The inset is the comparison between CTR and FF groups in the shoe-only condition. Dashed vertical lines indicate averaged phase boundaries. AUC values for gastrocnemius medialis activation during each gait phase: (b) contact, (c) foot flat, (d) propulsive, and (e) swing. CTR: control, FF: functional flat foot, AUC: area under the curve.

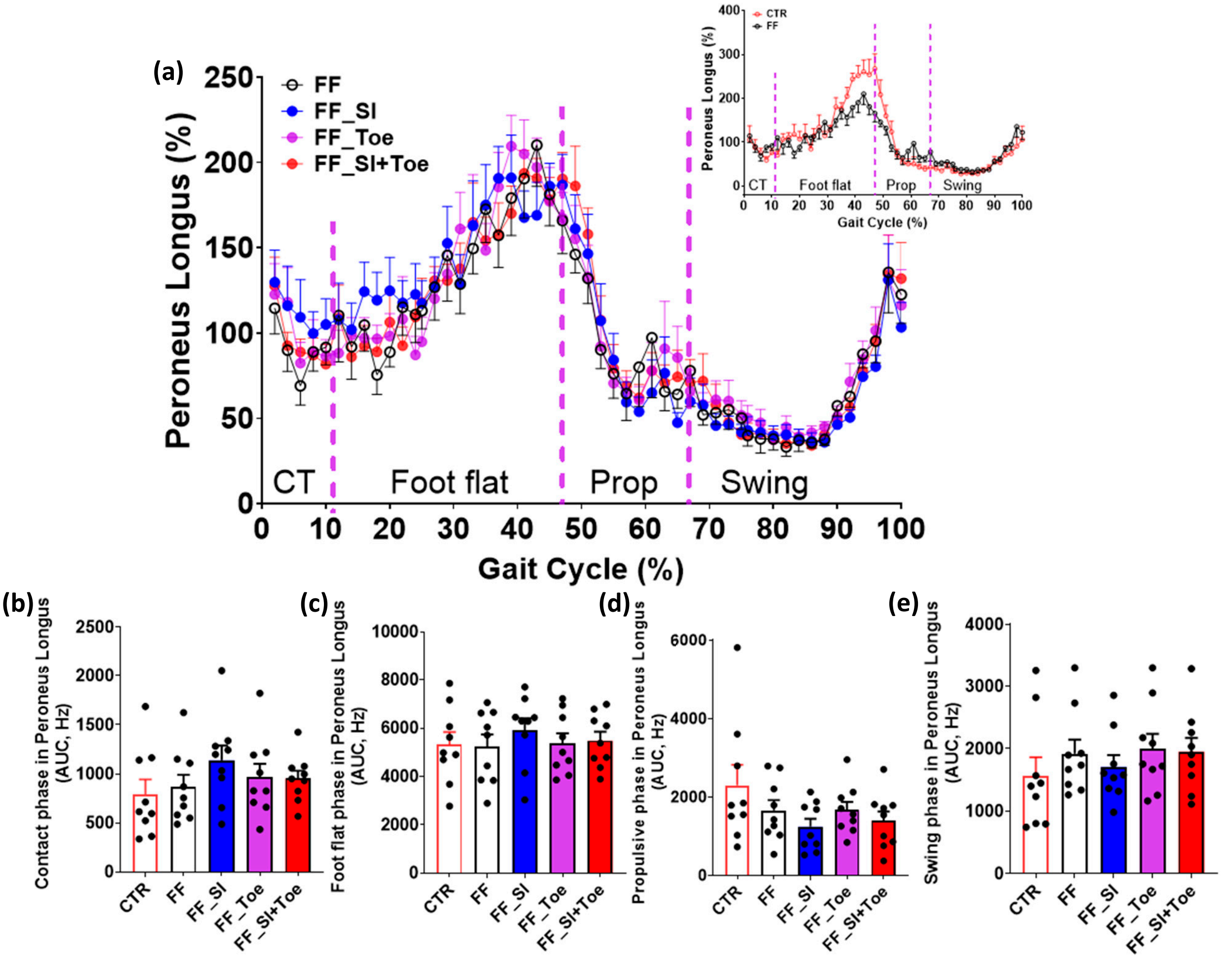

Supplementary 4. Peroneus longus muscle activation during level walking. (a) Averaged linear envelope EMG activity (% of mean EMG activities in each condition) across the gait cycle, segmented into contact, foot-flat, propulsive, and swing phases. The inset is the comparison between CTR and FF groups in the shoe-only condition. Dashed vertical lines indicate averaged phase boundaries. AUC values for peroneus longus activation during each gait phase: (b) contact, (c) foot flat, (d) propulsive, and (e) swing. CTR: control, FF: functional flat foot, AUC: area under the curve.
